# Three-dimensional spatial analysis of the temporomandibular joint in adult patients with Class II division 2 malocclusion before and after orthodontic treatment: a retrospective study

**DOI:** 10.1186/s12903-023-03210-9

**Published:** 2023-07-12

**Authors:** Jiajing Zheng, Yueying Zhang, Qiuyue Wu, Hua Xiao, Feifei Li

**Affiliations:** State Key Laboratory of Military Stomatology, National Clinical Research Center for Oral Diseases & Shaanxi Clinical Research Center for Oral Diseases, Department of Orthodontics, School of Stomatology, Air Force Medical University, Xi’an, 710032 Shaanxi People’s Republic of China

**Keywords:** Malocclusion, Temporomandibular joint, Orthodontic appliances, Cone-beam computed tomography

## Abstract

**Background:**

To investigate changes in the three-dimensional (3D) spatial morphology of the temporomandibular joint (TMJ) and condyle position in adult patients with Class II division 2 malocclusion using a 3D spatial measurement method and to investigate the similarities and differences in the effects of fixed appliance and clear aligner treatments on the TMJ.

**Methods:**

Cone-beam computed tomography (CBCT) data of 47 adult patients with Class II division 2 malocclusion (25, fixed appliance group; 22, clear aligner group) were collected before and after treatment. Mimics 21.0 was used to reconstruct the TMJ 3D model. Fourteen measurement items, such as the anterior, upper, and posterior joint spaces, were measured directly on the 3D model and compared.

**Results:**

Post-orthodontic treatment, the shape and position of the condyle changed in adult patients with Class II division 2 malocclusion. Reduction in the anterior joint space and increase in the posterior joint space after orthodontic treatment were significant in both fixed appliance and clear aligner treatments; the condyle moved forward to the center of the fossa. The superior joint space and depth of the glenoid fossa increased after clear aligner treatment, but there was no significant change after fixed appliance treatment.

**Conclusions:**

The condylar shape and position in patients with Class II division 2 malocclusion changed significantly post-treatment, indicating that the condyle undergoes adaptive reconstruction during orthodontic treatment in these patients. These results provide a reference for diagnosis, design of treatment plan, and monitoring of treatment in orthodontic clinics.

## Introduction

The objective of orthodontic treatment is balance, stability and aesthetics, namely, that orthodontists should focus on the health and balance of the patient's denture and temporomandibular joint (TMJ). The TMJ is one of the most complex joints in the body, and it also has the particularity of bilateral linkage, which needs attention in the orthodontic clinic [1]. Class II division 2 malocclusion is a common malocclusion, which is often caused by mandibular retrognathia, with the dental characteristics of severe lingual tipping of the upper anterior teeth, deep overbite, and distally related molars. This kind of malocclusion is multifactorial, influenced by both heredity and the environment. The lingual anterior teeth may impact the forward movement of the mandible, which may influence the morphology, position and health of the temporomandibular joint [2–4]. Condyle is the center of mandible growth and remodeling, so condyle morphology and position may be related to the formation and change of facial skeletal types [5–7]. Therefore, patients with this malocclusion are at risk for temporomandibular joint disorders (TMD) [8]. Some scholars have compared the occlusal characteristics of TMD patients with those of orthodontic patients and concluded that deep overbite is a risk factor for TMD [9]. Therefore, it is necessary to correct deep overbite as soon as possible in patients with Class II division 2 malocclusion to guarantee the development of the condyle and mandible.

Whether the TMJ can undergo adaptive modification by orthodontic treatment is a focus point in current research [10]. At present, most studies show that the position and shape of the condyle in these patients are different from those in skeletal class I patients [11–13]. The condyle is in a backward position, which provides space for guiding the mandible forward.

Clear aligner treatment is a gradually rising and popular orthodontic technology that has an occlusal splint effect caused by the thickness of the appliance patch [14]. However, there are few studies on the effects of clear aligner treatment on the TMJ of patients with Class II division 2 malocclusion.

Studies that compared the measurement of the TMJ by cone-beam computed tomography (CBCT) and panoramic radiographs showed that CBCT has a higher accuracy than traditional panoramic films [15]. At present, the two-dimensional measurement of the TMJ is more commonly used; CBCT data are imported into the measurement software, and the observation section is selected from the coronal, sagittal, and horizontal planes as the largest section area of the condyle; and the determination of the location of anatomical landmarks as well as the measurement of various indicators are carried out on that section. However, some authors have adopted another method, called three-dimensional (3D) spatial measurement. In this method, CBCT data are imported into the measurement software to reconstruct a 3D model, and the surveyor directly locates the anatomical landmarks and measures each index in the coronal, sagittal, and horizontal directions of the reconstructed 3D model. Compared with the two-dimensional measurement method, the three-dimensional spatial measurement is a more accurate method to use for analysis [16–19].

In this study, three-dimensional analysis of the TMJ before and after orthodontic treatment, and the differences and similarities between fixed appliance and clear aligner treatment on the TMJ were studied to provide a reference for orthodontic clinical practice.

## Methods

### Sample selection

This was a retrospective study with samples collected from our hospital. A total of 50 patients diagnosed with adult skeletal II Class II division 2 malocclusion from January 2018 to March 2023 were recruited as the study cohort, of which 3 patients dropped out due to problematic CBCT data. All the patients signed an informed consent form.

The inclusion criteria of the study subjects were as follows:

1. Age of first diagnosis > 18 years, secondary dentition
2. ANB > 4°, Severe lingual tipping of the upper anterior teeth, deep overbite III°, overjet < 2 mm, and distally related molars
3. Willingness to use a fixed appliance or a clear aligner for orthodontic treatment with non-extraction, non-guided mandible forward and being able to actively cooperate with the doctors
4. Post-orthodontic photos showed that all subjects’ teeth were neatly arranged, the occlusal relationship between canine teeth and molars was neutral, and the overbite and override of the anterior teeth reached normal level after orthodontic treatment.

The exclusion criteria were as follows:

1. Large area defects of the tooth, residual crown or root, tooth loss or congenital tooth loss
2. History of orthodontic treatment or other facial surgery
3. History of temporomandibular joint disorders; pain, flick, or murmur in the joint area or masticatory muscles
4. History of cyst or tumor surgery
5. History of cleft lip or cleft palate
6. History of systemic disease
7. Patients who were lost to follow-up

### Measurement methods and the measured items

All subjects underwent wide-field CBCT (NewTom AG, Marburg, Germany) head scans by radiologists at our hospital before and after orthodontic treatment. The voxel size of the CBCT is 150 μm and the grey scale is 16-bit. The subjects sat on the chair with their head position adjusted such that the Frankfort plane was parallel to the ground, the median sagittal plane consistent with the long axis of the fuselage, and the coronal plane perpendicular to the ground. During scanning, the posterior teeth on both sides were clenched and kept in their maximum intercuspation. The obtained images were stored on a computer or carved in the Digital Imaging and Communications in Medicine (DICOM) format.

**Fig. 1:**
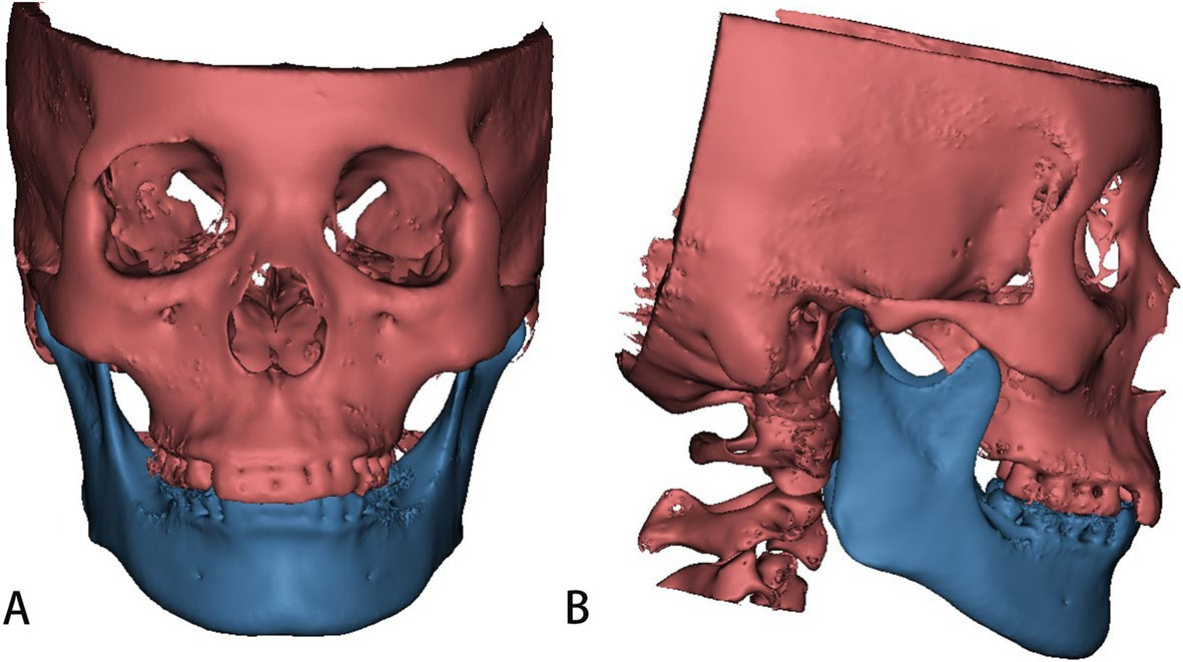
A three-dimensional model of craniomaxillofacial bones. **A**: Frontal; **B**: Lateral

**Fig. 2:**
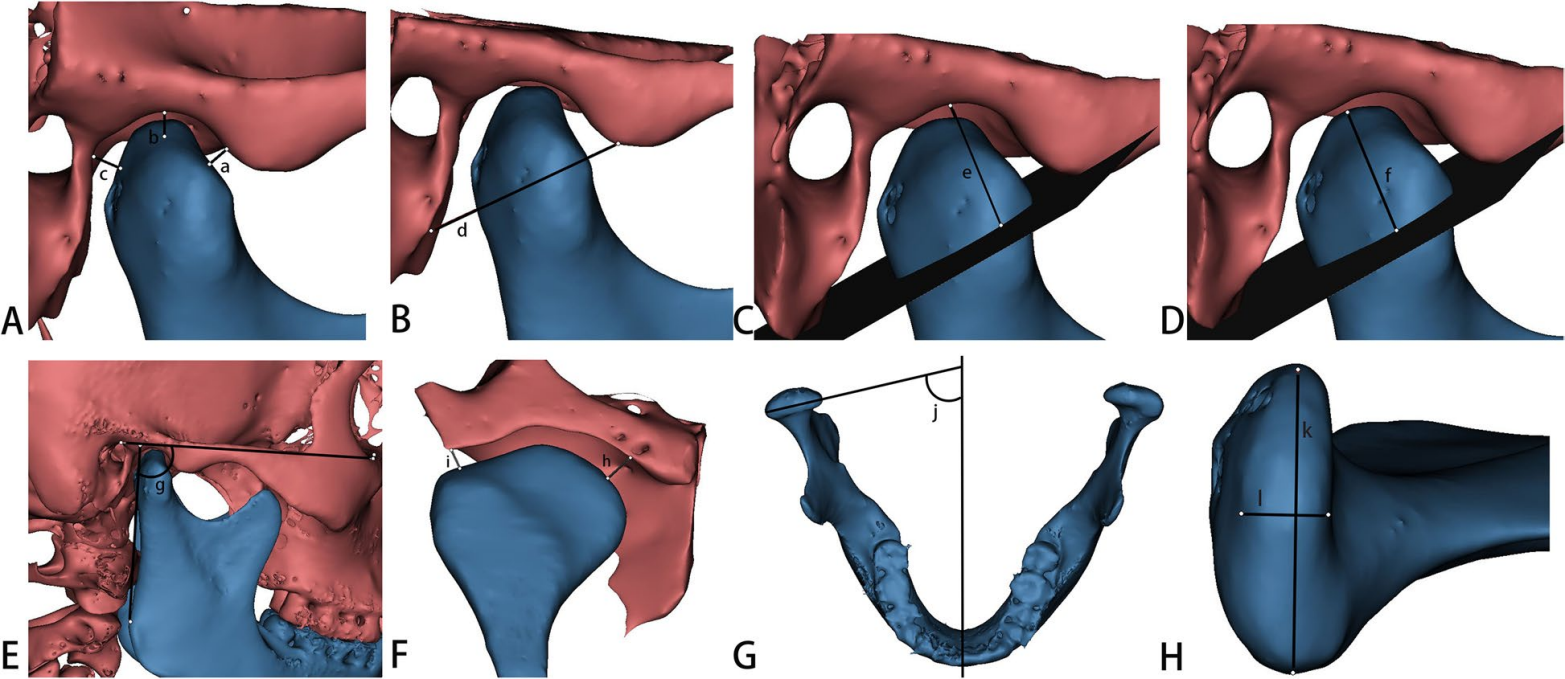
Measurement indices. **A**: a-anterior joint space, b-superior joint space, c-posterior joint space; **B**: d-width of the glenoid fossa; **C**: e-depth of the glenoid fossa; **D**: f-height of the condyle; **E**: g-sagittal condylar angle; **F**: h- interior joint space, i- exterior joint space; **G**: j-horizontal condylar angle; **H**: k- internal and external diameters of the condyle, l- anterior and posterior diameters of the condyle

**Fig. 3:**
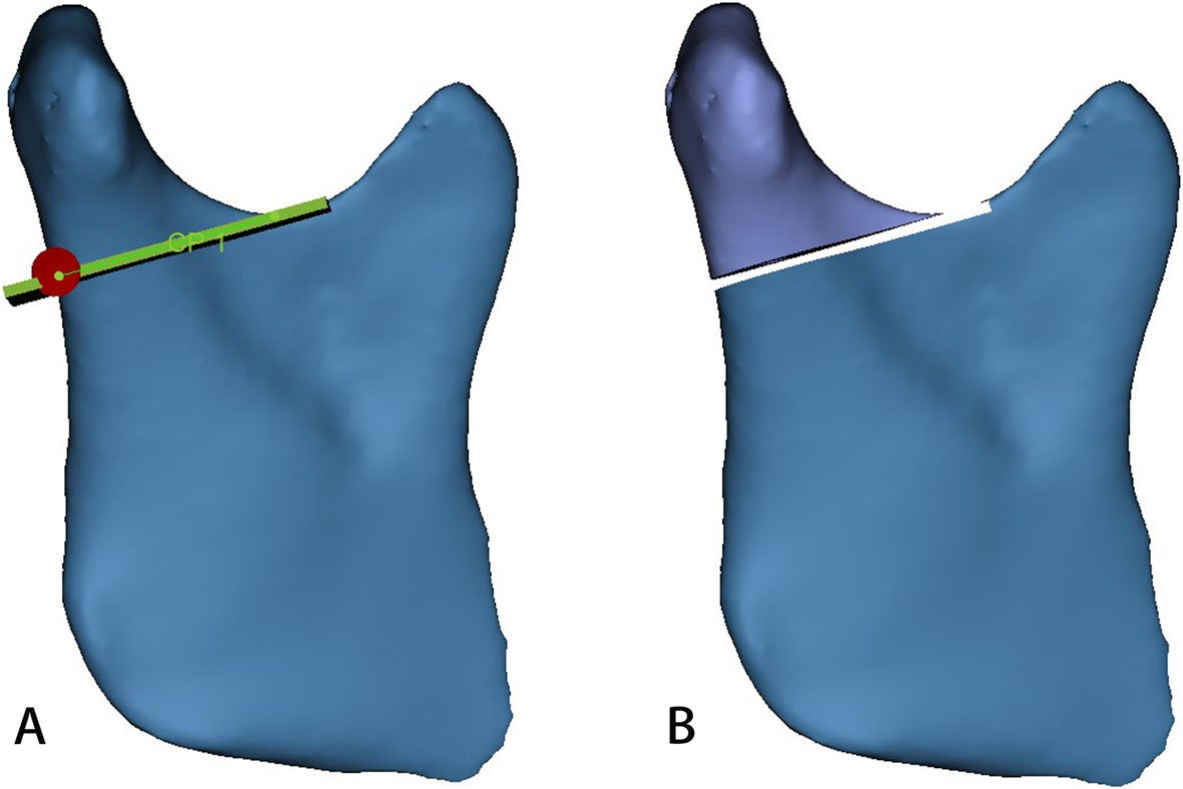
Measurement indices. **A**: in the sagittal direction, a section perpendicular to the ramus of the mandible is made through the lowest point of the mandible’s sigmoid notch; **B**: a 3D model of the condyle was segmented

The CBCT images of the subjects were imported into the Mimics software for 3D measurement (version 21.0; Materialise, Leuven, Belgium). The head position was adjusted so that the Frankfort plane was parallel to the horizontal plane, and the reference planes were determined as the Frankfort, midsagittal, and coronal planes. The gray threshold of the head and face bones was segmented, and a 3D model of the craniomaxillofacial bones was extracted (Fig. 1). The anatomical landmarks of the reconstructed 3D model were located in the horizontal, sagittal, and coronal directions, and the spatial measurement of each index was performed [16, 18, 20]. The measurement items are shown in Table 1 and Figs. 2 and 3.

**Table 1:** Definitions of measurement indices

Measurement index	Abbreviation	Definition
**Sagittal direction**		
Anterior joint space	AJS	The linear distance between the anterior point of the condyle and the posterior point of the joint nodule
Superior joint space	SJS	The linear distance between the uppermost point of the condyle and the uppermost point of the fossa
Posterior joint space	PJS	The linear distance between the last point of the condyle and the point of the posterior wall of the joint fossa
Width of the glenoid fossa	WGF	The linear distance from the nadir of the joint nodule to the point of the posterior concave process
Depth of the glenoid fossa	DGF	The perpendicular line from the uppermost point of the fossa to the nadir of the joint nodule and the point of the posterior process of the fossa
Height of the condyle	HC	The perpendicular line from the apex of the condyle to the nadir of the joint nodule and the point of the posterior process of the fossa
Sagittal condylar angle	SCA	The angle of intersection between the Frankfort plane and the tangential line of the mandibular ramus
**Coronal direction**		
Interior joint space	IJS	The linear distance from the innermost point of the condyle to the innermost point of the fovea
Exterior joint space	EJS	The linear distance from the outermost point of the condyle to the outermost point of the fovea
**Horizontal direction**		
Horizontal condylar angle	HCA	The intersection angle formed by the line "tip of the nose, septum of the nose, foramen magnum" and the extension line between the most lateral point of the condyle and the most medial point of the condyle
Internal and external diameters of the condyle	IEDC	The linear distance between the most lateral and medial points of the condyle
Anterior and posterior diameter of the condyle	APDC	The linear distance between the anterior and posterior points of the condyle
**Volume of the condyle**	**VC**	In the sagittal direction, a vertical line perpendicular to the mandibular ramus was made through the lowest point of the sigmoid notch of the mandible to segment the condyle, and the volume of the condyle was measured by MIMICS
**Surface area of the condyle**	**SC**	The surface area of the condyle was measured by MIMICS using the method described above

Condyle position was assessed according to Pullinger’s method, [21] LR = (posterior joint space − anterior joint space)/ (posterior joint space + anterior joint space) × 100%. If LR is > 12, the condyle is in the anterior portion of the fossa; -12 < LR < 12, the condyle is in the middle portion of the fossa; and LR < -12, the condyle is in the posterior portion of the fossa.

### Statistical methods

The G*power software (version 3.1; Universität Kiel) was used to calculate the sample size. Considering α = 0.05, β = 0.2, the t test for matched pairs, and an effect size of 0.8, the sample size was calculated as at least 15 patients in each group.

All 3D reconstruction and the determination of the location of anatomical landmarks of the TMJ were completed by the same researcher in a continuous period of time. SPSS software (version 25.0; IBM Corp., Armonk, NY, USA) was used for statistical analysis. The data were tested firstly for normality and homogeneity of variance using the Kolmogorov–Smirnov and Levene tests. The results showed that the data were consistent with normality and homogeneity of variance, and we use t-test for most of the statistical analysis in this study.

The chi-square test was used to compare the gender distribution between patients with fixed appliance and clear aligner treatments, an independent sample t-test was used to compare the ages and treatment duration between the two groups. A paired sample t-test was used to compare the differences between parameters of the left and right sides of the TMJ before and after treatment. It was also used to compare the differences between TMJ parameters before and after treatment. An independent sample t-test was used to compare the differences in TMJ measurement values before and after fixed appliance and clear aligner treatment. Differences were considered statistically significant at two-sided α = 0.05 and *P* < 0.05.

To verify the accuracy of the location and measurement results, 20 samples were randomly selected, and the measurements were repeated by another observer within 20 days after the initial measurement. Intraclass correlation coefficient (ICC) showed good agreement between the measurements (ICC > 0.95).

## Results

### General data

A total of 47 patients were finally included in the phase of measurement, including 25 patients in the fixed appliance group and 22 patients in the clear aligner group. There were no significant differences in sex, age and treatment duration between the two groups (Table 2), neither were significant differences found in the measurement values between the left and right sides of the joints in the two groups before and after treatment (Tables 3 and 4). There was no significant difference in the measurement values between the two groups before treatment (Table 5).

**Table 2:** Comparison of general conditions between the two appliance groups

Variable	n	Age (years old)	Treatment duration (months)	Sex	
				Male	Female
Fixed appliance group	25	22.80 ± 3.85	23.20 ± 1.85	12(48.00%)	13(52.00%)
Clear aligner group	22	23.18 ± 3.76	22.82 ± 2.13	10(45.45%)	12(54.55%)
*P*		0.733	0.514	0.861

**Table 3:** Comparison of the TMJ measurement values on the left and right sides before and after fixed appliance treatment ($\overline{x}\pm s,$ mm)

Variable	Before treatment			After treatment		
	Left	Right	*P*	Left	Right	*P*
AJS	2.57 ± 0.39	2.56 ± 0.43	0.742	2.38 ± 0.29	2.37 ± 0.28	0.722
SJS	3.03 ± 0.64	2.94 ± 0.64	0.054	2.96 ± 0.63	2.92 ± 0.66	0.377
PJS	1.94 ± 0.53	1.86 ± 0.50	0.138	2.21 ± 0.46	2.12 ± 0.37	0.119
WGF	25.59 ± 2.48	25.73 ± 2.55	0.658	25.95 ± 2.42	26.24 ± 2.55	0.338
DGF	11.19 ± 1.23	11.23 ± 0.89	0.844	11.21 ± 1.21	11.23 ± 0.76	0.925
HC	7.87 ± 1.24	8.05 ± 1.46	0.099	8.05 ± 1.24	8.20 ± 1.41	0.057
SCA (°)	75.73 ± 2.77	75.59 ± 2.59	0.728	75.76 ± 2.99	75.49 ± 2.56	0.539
IJS	2.84 ± 0.65	2.88 ± 0.70	0.423	2.83 ± 0.62	2.81 ± 0.69	0.764
EJS	2.68 ± 0.59	2.69 ± 0.49	0.797	2.67 ± 0.58	2.75 ± 0.46	0.259
HCA (°)	70.57 ± 2.83	70.21 ± 2.94	0.096	70.27 ± 2.99	70.52 ± 2.99	0.313
IEDC	16.49 ± 2.44	16.72 ± 2.41	0.199	17.03 ± 2.42	17.21 ± 2.53	0.223
APDC	6.82 ± 0.67	6.75 ± 0.76	0.446	7.11 ± 0.63	7.08 ± 0.75	0.677
VC (mm^3^)	1565.86 ± 336.09	1590.62 ± 281.81	0.436	1667.08 ± 318.17	1696.89 ± 281.27	0.286
SC (mm^2^)	1506.76 ± 332.10	1526.43 ± 277.33	0.565	1600.69 ± 331.22	1625.06 ± 299.85	0.447

*AJS* anterior joint space, *SJS* superior joint space, *PJS* posterior joint space, *WGF* width of the glenoid fossa, *DGF* depth of the glenoid fossa, *HC* height of the condyle, *SCA* sagittal condylar angle, *IJS* interior joint space, *EJS* exterior joint space, *HCA* horizontal condylar angle, *IEDC* internal and external diameters of the condyle, *APDC* anterior and posterior diameters of the condyle, *VC* volume of the condyle, *SC* surface area of the condyle

**Table 4:** Comparison of the TMJ measurement values on the left and right sides before and after clear aligner treatment ($\overline{x}\pm s,$ mm)

Variable	Before treatment			After treatment		
	Left	Right	*P*	Left	Right	*P*
AJS	2.55 ± 0.36	2.55 ± 0.35	0.753	2.35 ± 0.35	2.31 ± 0.32	0.254
SJS	2.85 ± 0.43	2.84 ± 0.46	0.759	3.52 ± 0.54	3.58 ± 0.61	0.052
PJS	2.01 ± 0.40	1.98 ± 0.34	0.406	2.36 ± 0.41	2.34 ± 0.37	0.674
WGF	24.85 ± 2.11	24.81 ± 2.12	0.736	24.87 ± 2.12	24.83 ± 1.94	0.735
DGF	11.35 ± 0.87	11.37 ± 0.76	0.884	11.74 ± 0.64	11.66 ± 0.77	0.601
HC	7.71 ± 0.79	7.83 ± 0.73	0.372	7.78 ± 0.65	7.88 ± 0.67	0.386
SCA (°)	75.21 ± 2.20	74.38 ± 2.42	0.063	74.12 ± 2.61	74.37 ± 2.50	0.152
IJS	2.92 ± 0.49	2.78 ± 0.52	0.074	2.90 ± 0.44	2.81 ± 0.48	0.063
EJS	2.83 ± 0.42	2.85 ± 0.42	0.359	2.85 ± 0.37	2.87 ± 0.34	0.537
HCA (°)	71.68 ± 3.29	71.45 ± 3.56	0.420	71.90 ± 2.99	71.57 ± 3.42	0.233
IEDC	16.71 ± 1.42	16.84 ± 1.41	0.211	17.23 ± 1.43	17.29 ± 1.42	0.595
APDC	7.01 ± 0.86	7.04 ± 0.89	0.760	7.46 ± 0.89	7.41 ± 0.95	0.408
VC (mm^3^)	1574.19 ± 232.32	1574.79 ± 236.78	0.954	1677.02 ± 237.88	1686.57 ± 232.18	0.348
SC (mm^2^)	1505.39 ± 236.72	1500.09 ± 215.89	0.586	1608.93 ± 248.93	1603.46 ± 235.83	0.550

*AJS* anterior joint space, *SJS* superior joint space, *PJS* posterior joint space, *WGF* width of the glenoid fossa, *DGF* depth of the glenoid fossa, *HC* height of the condyle, *SCA* sagittal condylar angle, *IJS* interior joint space, *EJS* exterior joint space, *HCA* horizontal condylar angle, *IEDC* internal and external diameters of the condyle, *APDC* anterior and posterior diameters of the condyle, *VC* volume of the condyle, *SC* surface area of the condyle

**Table 5:** Comparison of the TMJ measurements between the two groups before orthodontic treatment ($\overline{x}\pm s$, mm)

Variable	Fixed appliance	Clear aligner	t	*P*
AJS	2.57 ± 0.41	2.55 ± 0.35	0.199	0.843
SJS	2.98 ± 0.63	2.85 ± 0.44	1.172	0.244
PJS	1.90 ± 0.51	1.99 ± 0.37	-1.008	0.316
WGF	25.66 ± 2.49	24.83 ± 2.09	1.746	0.084
DGF	11.21 ± 1.06	11.36 ± 0.80	-0.767	0.445
HC	7.96 ± 1.34	7.77 ± 0.75	0.845	0.400
SCA (°)	75.66 ± 2.66	74.79 ± 2.32	1.681	0.096
IJS	2.86 ± 0.67	2.85 ± 0.50	0.088	0.930
EJS	2.68 ± 0.54	2.85 ± 0.42	-1.615	0.110
HCA (°)	70.39 ± 2.86	71.56 ± 3.39	-1.818	0.072
IEDC	16.61 ± 2.40	16.78 ± 1.40	-0.419	0.676
APDC	6.79 ± 0.71	7.03 ± 0.87	-1.478	0.143
VC (mm^3^)	1578.24 ± 307.22	1574.49 ± 231.82	0.066	0.947
SC (mm^2^)	1516.59 ± 302.97	1502.74 ± 223.91	0.249	0.804

*AJS* anterior joint space, *SJS* superior joint space, *PJS* posterior joint space, *WGF* width of the glenoid fossa, *DGF* depth of the glenoid fossa, *HC* height of the condyle, *SCA* sagittal condylar angle, *IJS* interior joint space, *EJS* exterior joint space, *HCA* horizontal condylar angle, *IEDC* internal and external diameters of the condyle, *APDC* anterior and posterior diameters of the condyle, *VC* volume of the condyle, *SC* surface area of the condyle

### Morphological and positional changes of the TMJ

After fixed appliance treatment, the anterior joint space and interior joint space decreased, while the width of the glenoid fossa, height of the condyle, internal and external diameters of the condyle, anterior and posterior diameters of the condyle, the condyle volume and the condyle surface area increased. The average volume and surface area of the condyle increased by approximately 103.75 mm^3^ and 96.28 mm^2^ respectively. The above results were statistically significant (Table 6).

**Table 6:** Comparison of the TMJ measurement values before and after treatment in two appliance groups ($\overline{x}\pm s,$ mm)

Variable	Fixed appliance group			Clear aligner group		
	Before treatment	After treatment	*P*	Before treatment	After treatment	*P*
AJS	2.57 ± 0.41	2.38 ± 0.28	< 0.001^*^	2.55 ± 0.35	2.33 ± 0.33	< 0.001^*^
SJS	2.98 ± 0.63	2.94 ± 0.64	0.062	2.85 ± 0.44	3.55 ± 0.57	< 0.001^*^
PJS	1.90 ± 0.51	2.16 ± 0.41	< 0.001^*^	1.99 ± 0.37	2.35 ± 0.38	< 0.001^*^
WGF	25.66 ± 2.49	26.09 ± 2.47	< 0.001^*^	24.83 ± 2.09	24.85 ± 2.01	0.760
DGF	11.21 ± 1.06	11.22 ± 1.00	0.808	11.36 ± 0.80	11.70 ± 0.70	< 0.001^*^
HC	7.96 ± 1.34	8.13 ± 1.32	0.001^*^	7.77 ± 0.75	7.83 ± 0.65	0.181
SCA (°)	75.66 ± 2.66	75.63 ± 2.76	0.792	74.79 ± 2.32	74.74 ± 2.55	0.740
IJS	2.86 ± 0.67	2.82 ± 0.65	0.036^*^	2.85 ± 0.50	2.86 ± 0.46	0.857
EJS	2.68 ± 0.54	2.71 ± 0.52	0.252	2.85 ± 0.42	2.86 ± 0.35	0.461
HCA (°)	70.39 ± 2.86	70.40 ± 2.96	0.975	71.56 ± 3.39	71.73 ± 3.18	0.095
IEDC	16.61 ± 2.40	17.12 ± 2.45	< 0.001^*^	16.78 ± 1.40	17.26 ± 1.41	< 0.001^*^
APDC	6.79 ± 0.71	7.09 ± 0.69	< 0.001^*^	7.03 ± 0.87	7.44 ± 0.91	< 0.001^*^
VC (mm^3^)	1578.24 ± 307.22	1681.99 ± 297.59	< 0.001^*^	1574.49 ± 231.82	1681.79 ± 232.35	< 0.001^*^
SC (mm^2^)	1516.59 ± 302.97	1612.87 ± 312.93	< 0.001^*^	1502.74 ± 223.91	1606.20 ± 239.65	< 0.001^*^

*AJS* anterior joint space, *SJS* superior joint space, *PJS* posterior joint space, *WGF* width of the glenoid fossa, *DGF* depth of the glenoid fossa, *HC* height of the condyle, *SCA* sagittal condylar angle, *IJS* interior joint space, *EJS* exterior joint space, *HCA* horizontal condylar angle, *IEDC* internal and external diameters of the condyle, *APDC* anterior and posterior diameters of the condyle, *VC* volume of the condyle, *SC* surface area of the condyle

^*^P < 0.05

After clear aligner treatment, anterior joint space decreased, while superior joint space, posterior joint space, depth of the glenoid fossa, internal and external diameters of the condyle, anterior and posterior diameters of the condyle, volume and surface area of the condyle increased in adult patients with Class II division 2 malocclusion, with the average volume and surface area of the condyle increasing by approximately 107.30 mm^3^ and 103.46 mm^2^, respectively. The above results were statistically significant (Table 6).

Regardless of whether fixed appliance or clear aligner treatment had been applied, the sagittal position of the condyle in the fossa changed after treatment.

According to Pullinger’s method, [21] the condyle positions of the two groups of subjects before and after orthodontic treatment were calculated. Before fixed appliance treatment, about 68% patients’ condyles were in the posterior portion of the articular fossa, while only 24% of the patients’ condyles were in the posterior portion after fixed appliance treatment. The condyles of 22 patients with Class II division 2 malocclusion, were posterior to the fossa in 61.36% before clear aligner treatment and were central to the fossa in 86.36% after clear aligner treatment; changes in the condylar position were statistically significant (Table 7).

**Table 7:** Comparison of the condylar position in the articular fossa before and after orthodontic treatment (n, %)

	Fixed appliance				Clear aligner			
Variable	Before treatment	After treatment	χ2	*P*	Before treatment	After treatment	χ2	*P*
n	50	50	19.485	< 0.001^*^	44	44	21.382	< 0.001^*^
Condyle in anteposition	0	0			0	0		
Condyle in the middle	16(32.00%)	38(76.00%)			17(38.64%)	38(86.36%)		
Condyle in retroposition	34(68.00%)	12(24.00%)			27(61.36%)	6(13.64%)		

^*^P < 0.05

### Fixed appliance and clear aligner treatments on the TMJ

The results of the comparison of TMJ-related structural changes after fixed appliance and clear aligner treatments were as follows: there were significant differences in the measurement values of the superior joint space, fossa width and fossa depth before and after treatment. The superior joint space of adult patients with Class II division 2 malocclusion increased after clear aligner treatment, but there were no significant changes after fixed appliance treatment (Table 8).

**Table 8:** Comparison of the changes after fixed appliance and clear aligner treatments in TMJ measurement values ($\overline{x}\pm s$, mm)

Variable	Fixed appliance	Clear aligner	t	*P*
AJS	-0.19 ± 0.27	-0.23 ± 0.20	0.703	0.484
SJS	-0.04 ± 0.15	0.70 ± 0.40	-11.658	< 0.001^*^
PJS	0.26 ± 0.24	0.36 ± 0.23	-1.913	0.059
WGF	0.43 ± 0.47	0.02 ± 0.51	4.052	< 0.001^*^
DGF	0.01 ± 0.26	0.34 ± 0.34	-5.295	< 0.001^*^
HC	0.17 ± 0.33	0.06 ± 0.29	1.673	0.098
SCA (°)	-0.04 ± 0.96	-0.05 ± 1.02	0.074	0.941
IJS	-0.04 ± 0.13	0.01 ± 0.17	-1.4	0.165
EJS	0.03 ± 0.16	0.02 ± 0.15	0.311	0.756
HCA (°)	0.01 ± 1.06	0.17 ± 0.67	-0.899	0.371
IEDC	0.51 ± 0.32	0.48 ± 0.35	0.395	0.693
APDC	0.31 ± 0.26	0.41 ± 0.36	-1.525	0.131
VC (mm^3^)	103.75 ± 65.00	107.30 ± 40.75	-0.322	0.749
SC (mm^2^)	96.28 ± 56.54	103.46 ± 60.46	-0.595	0.554

*AJS* anterior joint space, *SJS* superior joint space, *PJS* posterior joint space, *WGF* width of the glenoid fossa, *DGF* depth of the glenoid fossa, *HC* height of the condyle, *SCA* sagittal condylar angle, *IJS* interior joint space, *EJS* exterior joint space, *HCA* horizontal condylar angle, *IEDC* internal and external diameters of the condyle, *APDC* anterior and posterior diameters of the condyle, *VC* volume of the condyle, *SC* surface area of the condyle

^*^P < 0.05

## Discussion

This study focused on the structural characteristics of the TMJ in patients with Class II division 2 malocclusion. The three-dimensional spatial measurement method was used to directly measure the TMJ on the reconstructed 3D model. The differences and similarities between fixed appliance and clear aligner treatments on the TMJ were compared for a more accurate assessment of the changes in the TMJ due to orthodontic treatment in patients with Class II division 2 malocclusion, which can provide guidance for the formulation of orthodontic clinical treatment plans and the selection of treatment appliances.

Most previous studies on the TMJ used X-ray cephalometry films, panoramic radiographs, or CBCT two-dimensional measurement methods in which the TMJ structure was measured in a cross-section. These methods can only measure one section of the distance between two points and are unable to measure the three-dimensional distance between two points, ignoring the influence of depth on the measured value. The measurements tend to be smaller than those obtained using the three-dimensional spatial measurement method [16, 19]. Therefore, the three-dimensional spatial measurement method was adopted in this experiment to measure TMJ.

We found that in a large proportion of adult patients with Class II division 2 malocclusion the condyle was retro-displaced, and that the condyle moved forward to the center of the fossa after orthodontic treatment. Moreover, width and depth of the articular fossa, anterior and posterior diameters, internal and external diameters, volume, and surface area of the condyle increased to a certain extent, indicating that the condyles of the patients were not only changed in position, but also in morphology. Orthodontic treatment may lead to adaptive reconstruction of condyles in adult patients with Class II division 2 malocclusion. At the same time, the articular fossa of the patients also underwent corresponding remodeling to adapt to the changes of condyles.

Many scholars have concluded that after functional or fixed appliance treatment, the mandible in the retrograde position is released and moves forward in patients with Class II division 2 malocclusion [22, 23]. In addition, panoramic radiography, CBCT, and MRI revealed that the condyle underwent adaptive remodeling after treatment, and new bone was formed on the surface of the condyle. However, Feres et al. found no significant difference in the sagittal position of the condyle in patients with Class I and Class II division 2 malocclusions. The authors denied the claim that the mandible moves forward after the release of the deep overbite [24]. However, their study inferred that orthodontic treatment did not change the condylar position based on cross-sectional findings; this conclusion requires further experimental verification. At the same time, there are few studies on the effects of clear aligner treatment on the TMJ. Shen used a sagittal-guided twin-block (SGTB) appliance designed to treat patients with Class II division 1 malocclusion. He observed that the SGTB, as an invisible functional appliance, can cause adaptive remodeling of the condyle [25].

Therefore, it can be inferred from the above results that regardless of which appliance is used in the treatment of patients with Class II division 2 malocclusion, the mandible will be released after correction of the deep overbite, and the position of the condyle will also change, along with adaptive reconstruction, which improves stability after treatment.

In general, when a fixed appliance is used for the treatment of patients with Class II division 2 malocclusion, the maxillary appliance is usually bonded first, and a flat bite plate is worn to open the bite [26]. After the bite is opened, the lower appliance is bonded. Because clear aligner encircles the entire tooth crown and the patch has a certain thickness, it is more conducive to opening the occlusion and releasing the mandible. Moreover, the maxilla and mandible can be corrected simultaneously, which improves the efficiency of treatment and enhances stability during the maintenance stage [27].

The thickness of clear aligner is approximately 0.77 mm, and the total thickness of the upper and lower appliances is approximately 1.5 mm [28]; if an occlusal splint is fitted, it will be thicker. When the upper and lower teeth are occluded with clear aligners, the position of the mandible changes, which may affect the muscle strength of the face and neck. However, there are few studies on the effects of clear aligners on the structure of the TMJ, and whether long-term use of clear aligners will change the joint space and affect the position of the condyle requires further study.

The results of this study showed that the superior joint space and depth of the glenoid fossa increased after clear aligner treatment in adult patients with Class II division 2 malocclusion, but there was no significant change after fixed appliance treatment. It is speculated that this result is due to the occlusal splint effect of the clear aligner. Because the diaphragm of the clear aligner has a certain thickness, when the patient wears it for a long time, the occlusal space will be opened to a certain extent and the superior joint space will also increase with the change in the position of the mandible [29]. However, because the hardness of clear aligner is different from that of the resin materials used in the past, the influences on the TMJ cannot be inferred from previous studies, and it is necessary to re-evaluate them. Several studies have shown that wearing a transparent wraparound retainer of a material similar to that of the clear aligner for 3–6 months does not change the joint space and does not cause obvious irreversible displacement of the mandible position [30]. At present, there is no evidence of negative effects of the clear aligner on the TMJ in patients without craniomandibular musculature disorders [31]; however, it is too early to conclude that there are no additional effects of clear aligner treatment on the TMJ. According to the results of the current study, the increase in the superior joint space and depth of the glenoid fossa did not cause any discomfort, which can be regarded as a result of the adaptive reconstruction of the condyle to the occlusal splint effect.

This is a retrospective clinical study, and there may have been some uncontrollable confounding factors that affected the experimental results to some extent. It would be helpful to set untreated patients as a control group to eliminate interference, but it is difficult for us to obtain untreated patients’ imaging data due to ethical reasons, that is a limitation of this study. Therefore, adult patients were selected for the study in order to exclude the influence of condyle growth on the experimental results as much as possible. Meanwhile, this study only included non-extraction orthodontic treatments, as most of the patients with Class II division 2 malocclusion underwent this kind of treatment in the orthodontic clinic. Confirmation of these results in extraction orthodontic treatment awaits further studies; however, in such cases confounding factors are more difficult to control and would pose additional difficulties in terms of measurement and analysis.

At present, we are conducting a prospective longitudinal study on the motion trajectory and changes in the morphology and position of the TMJ in adolescent patients with Class II division 1 malocclusion who are undergoing functional mandibular advancement, to evaluate whether orthodontic treatment can move the condyle to a comfortable position. Meanwhile, a long-term observation of the stability of the mandible position in patients who have completed this kind of treatment is scheduled.

## Conclusions

1. Volume and surface area of the condyles increased in most adult patients with Class II division 2 malocclusion under fixed appliance or clear aligner treatment, indicating that the condyles may have undergone adaptive remodeling.
2. The condyle was displaced forward to the center of the glenoid fossa after fixed appliance or clear aligner treatment in most adult patients with Class II division 2 malocclusion, indicating that orthodontic treatment may provide more adequate space for mandible and condyle after correcting the deep overbite of anterior teeth, and the forward movement of mandible is accompanied by the adaptive reconstruction of the condyle.
3. Compared with the effects of clear aligner and fixed appliance treatments on the TMJ of adult patients with Class II division 2 malocclusion, the superior joint space and glenoid fossa depth of patients in the clear aligner appliance group increased, while no significant changes were found in these two indexes in the fixed appliance group, which may be due to patients being affected by the occlusal splint effect of wearing clear aligners for a long time.

## Data Availability

The datasets used and analysed during the current study available from the corresponding author on reasonable request.

## Abbreviations

3D: Three-dimensional
TMJ: Temporomandibular joint
CBCT: Cone-beam computed tomography
TMD: Temporomandibular joint disorders
DICOM: Digital imaging and communications in medicine
SGTB: Sagittal-guided twin-block
